# Comment on “Polydeoxyribonucleotide Exerts Therapeutic Effect by Increasing VEGF and Inhibiting Inflammatory Cytokines in Ischemic Colitis Rats”

**DOI:** 10.1155/2023/9803930

**Published:** 2023-07-03

**Authors:** Donatella Di Fabrizio, Salvatore Arena, Pietro Impellizzeri, Carmelo Romeo

**Affiliations:** Department of Human Pathology of Adult and Childhood “Gaetano Barresi”, Unit of Pediatric Surgery, University of Messina, Messina, Italy

We read and highly appreciated the interesting article by Kim et al. [1], focusing on the potential therapeutic effects of polydeoxyribonucleotide (PDRN) as an ischemic colitis medical treatment. The colon is particularly susceptible to hypoperfusion and critical low-flow state precipitate to an often innate preexisting less developed microvasculature [2]. As a compensatory mechanism, in the first stage of the colonic hypoxic state, vascular endothelial growth factor (VEGF) and hypoxia-inducible factor-1*α* (HIF-1*α*) are induced, and they paracrinally act to preserve tissue from ischemic damage. By attempting to counteract the hypoxic condition, activation of adenosine A_2A_ receptors (A_2A_R) occurs in a cooperative fashion with hypoxia, modulating the anti-inflammatory pathway and stimulating VEGF release [3]. PDRN, a specific ligand of A_2A_R, further enhances the VEGF expression, triggering a downregulation of inflammatory cytokines and an inhibition of both intrinsic and extrinsic apoptotic machineries [4–5]. Moreover, PDRN acts with a “salvage pathway” through the cell internalization of PDRN-derived purine and pyrimidine, mostly where *de novo* synthesis of DNA is severely impaired by ischemic condition, allowing a rapid tissue recovery [6]. This bioactive natural compound turns out to have relevant therapeutic effects in a range of pathological conditions, for its tissue repairing, anti-ischemic, and anti-inflammatory properties [6].

In Kim et al. [1], the authors documented a PDRN ability in increasing VEGF expression, reducing the histological damage, downregulating anti-inflammatory responses, and modulating apoptosis through the interaction with adenosine A_2A_R. In this regard, the effects of PDRN in the experimental model of ischemic colitis are abolished using DMPX, a specific adenosine A_2A_R antagonist. This observation minimizes the involvement of the “salvage pathway” in the mechanism of action of PDRN in ischemic colitis. Furthermore, considering the involvement of VEGF in the activation of adenosine A_2A_R, we believe it could be advantageous to demonstrate the effect of PDRN on neoangiogenesis and microvessel density, which may result in an augmented oxygen supply and a subsequent balance of the apoptotic mechanism [3].

To date, one of the most important ongoing clinical PDRN applications is wound healing, in which it increases reepithelialization and tissue granulation, increases the production of VEGF and blood vessels, and reduces the infiltration of inflammatory cells and scar size [7, 8]. Furthermore, it has also been used to reduce neuropathic pain and brain damage, to heal tendon injuries, to contrast the effect of estrogen deficiency-induced osteoporosis, and with intratracheal instillation to reduce lung inflammation and injury [9].

In conclusion, we believe that the data of Kim et al. [1] are remarkable, and considering the safe profile of PDRN also in humans, it could be useful to attempt a clinical randomized trial to verify the properties of this promising compound.

## References

[1] Kim S. E., Ko I. G., Jin J. J. (2020). Polydeoxyribonucleotide exerts therapeutic effect by increasing VEGF and inhibiting inflammatory cytokines in ischemic colitis rats. *BioMed Research International*.

[2] Theodoropoulou A., Koutroubakis I. E. (2008). Ischemic colitis: clinical practice in diagnosis and treatment. *World Journal of Gastroenterology*.

[3] Arena S., Minutoli L., Arena F. (2012). Polydeoxyribonucleotide administration improves the intra-testicular vascularization in rat experimental varicocele. *Fertility and Sterility*.

[4] Minutoli L., Arena S., Bonvissuto G. (2011). Activation of adenosine A_2A_ receptors by polydeoxyribonucleotide increases vascular endothelial growth factor and protects against testicular damage induced by experimental varicocele in rats. *Fertility and Sterility*.

[5] Minutoli L., Antonuccio P., Squadrito F. (2012). Effects of polydeoxyribonucleotide on the histological damage and the altered spermatogenesis induced by testicular ischaemia and reperfusion in rats. *International Journal of Andrology*.

[6] Squadrito F., Bitto A., Irrera N. (2017). Pharmacological activity and clinical use of PDRN. *Frontiers in Pharmacology*.

[7] Belmontesi M. (2020). Polydeoxyribonucleotide for the improvement of a hypertrophic retracting scar-an interesting case report. *Journal of Cosmetic Dermatology*.

[8] Lee J. H., Han J. W., Byun J. H., Lee W. M., Kim M. H., Wu W. H. (2018). Comparison of wound healing effects between Oncorhynchus keta-derived polydeoxyribonucleotide (PDRN) and Oncorhynchus mykiss-derived PDRN. *Archives of Craniofacial Surgery*.

[9] Kim T. H., Heo S. Y., Oh G. W., Heo S. J., Jung W. K. (2021). Applications of marine organism-derived polydeoxyribonucleotide: its potential in biomedical engineering. *Marine Drugs*.

